# Identification of a glycosylphosphatidylinositol anchor-modifying β1-3 galactosyltransferase in *Trypanosoma brucei*

**DOI:** 10.1093/glycob/cwu131

**Published:** 2014-12-02

**Authors:** Luis Izquierdo, Alvaro Acosta-Serrano, Angela Mehlert, Michael AJ Ferguson

**Affiliations:** 2 Division of Biological Chemistry and Drug Discovery, The College of Life Sciences, University of Dundee, Dundee DD1 5EH, UK; 3 Barcelona Centre for International Health Research, CRESIB, Hospital Clínic-Universitat de Barcelona, Barcelona 08036, Spain; 4 Department of Parasitology; 5 Department of Vector Biology, Liverpool School of Tropical Medicine, Liverpool L3 5QA, UK

**Keywords:** galactosyltransferase, GPI-anchor, procyclic form, TbGT3, *Trypanosoma brucei*

## Abstract

*Trypanosoma brucei* is the causative agent of human African sleeping sickness and the cattle disease nagana. * Trypanosoma brucei* is dependent on glycoproteins for its survival and infectivity throughout its life cycle. Here we report the functional characterization of *TbGT3*, a glycosyltransferase expressed in the bloodstream and procyclic form of the parasite. Bloodstream and procyclic form *TbGT3* conditional null mutants were created and both exhibited normal growth under permissive and nonpermissive conditions. Under nonpermissive conditions, the normal glycosylation of the major glycoprotein of bloodstream form *T. brucei*, the variant surface glycoprotein and the absence of major alterations in lectin binding to other glycoproteins suggested that the major function of TbGT3 occurs in the procyclic form of the parasite. Consistent with this, the major surface glycoprotein of the procyclic form, procyclin, exhibited a marked reduction in molecular weight due to changes in glycosylphosphatidylinositol (GPI) anchor side chains. Structural analysis of the mutant procyclin GPI anchors indicated that *TbGT3* encodes a UDP-Gal: β-GlcNAc-GPI β1-3 Gal transferase. Despite the alterations in GPI anchor side chains, *TbGT3* conditional null mutants remained infectious to tsetse flies under nonpermissive conditions.

## Introduction

The tsetse-transmitted *Trypanosoma brucei* group of parasites cause Human African Trypanosomiasis and nagana in cattle and constitute a serious health problem for people and livestock in 36 countries of sub-Saharan Africa. * Trypanosoma brucei* exists in the mammalian host as the bloodstream form trypomastigote and in the midgut of the tsetse fly vector (*Glossina* sp.) as the procyclic form. The bloodstream form of the parasite in the mammalian host is covered by a glycosylphosphatidylinositol (GPI)-anchored coat of 5 × 10^6^ variant surface glycoprotein (VSGs) homodimers and it evades the immune system by periodically replacing the existing VSG coat by a different one, a phenomenon known as antigenic variation (Cross 1996). Depending on the VSG variant GPI anchors contain side chains of 0–6 Gal residues (Ferguson et al. 1988) and between 1 and 3 N-linked glycans. The latter can be of the conventional oligomannose, paucimannose or complex types (Zamze et al. 1990, 1991; Jones et al. 2005; Manthri et al. 2008; Izquierdo, Schulz, et al. 2009).

When bloodstream form parasites are ingested by the tsetse fly, they differentiate to the procyclic form in the insect midgut. During this process, the VSG coat is replaced by a mix of GPI-anchored procyclins, a non-GPI surface coat (Guther et al. 2006) as well as free GPIs (Lillico et al. 2003; Vassella et al. 2003; Nagamune et al. 2004; Roper et al. 2005). Procyclins are characterized by internal dipeptide (EP) or pentapeptide (GPEET) repeats which confer a rod-like structure to the protein (Roditi et al. 1989; Treumann et al. 1997).

* Trypanosoma brucei* strain 427, the strain used in this study, contains (per diploid genome): two copies of the GPEET1 gene encoding six GPEET repeats; one copy each of the EP1-1 and EP1-2 genes, encoding EP1 procyclins with 30 and 25 EP repeats, respectively; two copies of the EP2-1 gene, encoding EP2 procyclin with 25 EP repeats; and two copies of the EP3-1 gene, encoding EP3 procyclin with 22 EP repeats (Acosta-Serrano et al. 1999). The EP1 and EP3 procyclins contain a single N-glycosylation site, occupied exclusively by a conventional triantennary oligomannnose Man_5_GlcNAc_2_ oligosaccharide, at the N-terminal side of the EP repeat domain (Treumann et al. 1997; Acosta-Serrano et al. 1999). Either EP2 or GPEET procyclins are N-glycosylated and GPEET1 procyclin is phosphorylated on six of seven Thr residues (Mehlert et al. 1999; Schlaeppi et al. 2003). Both GPEET and EP procyclins contain indistinguishable GPI membrane anchors. These are the largest and most complex anchors known and they are characterized by the presence of large poly-disperse-branched *N*-acetyllactosamine (Galβ1-4GlcNAc) and lacto-*N*-biose (Galβ1-3GlcNAc)-containing side chains that can be capped with α2-3-linked sialic acid residues (Ferguson et al. 1993; Treumann et al. 1997). Sialic acids are transferred from serum sialoglycoconjugates to terminal β-galactose residues by the action of a cell surface trans-sialidase enzyme (Engstler et al. 1993; Pontes de Carvalho et al. 1993; Montagna et al. 2002,  2006) and trans-sialylation of surface components plays a role in the successful colonization of the tsetse fly (Nagamune et al. 2004). In addition, it has been postulated that the branched side chains of the anchor form a dense glycocalyx that contributes to the protective function of the coat against digestive enzymes in the fly midgut (Acosta-Serrano et al. 2001).

As well as the aforementioned major GPI-anchored surface molecules, *T. brucei* expresses numerous other GPI-anchored and transmembrane glycoproteins at the cell surface, in the flagellar pocket and in the intracellular endosomal–lysosomal system, some of which are lifecycle stage- or display lifecycle stage-specific glycosylation differences. For example, the transmembrane invariant surface glycoproteins ISG65 and ISG75 (Ziegelbauer and Overath 1992) and the GPI-anchored flagellar pocket ESAG6/ESAG7 heterodimeric transferrin receptors (Steverding et al. 1995; Steverding 2000; Mehlert and Ferguson 2007; Mehlert et al. 2012) are specific to the bloodstream lifecycle stage while the major lysosomal glycoprotein is common to bloodstream and procyclic stages but contains complex *N*-glycans only in the bloodstream stage (Kelley et al. 1995). This control of stage-specific glycosylation probably resides primarily at the level of oligosaccharyltransferase expression (Izquierdo, Schulz, et al. 2009).

The survival strategies of protozoan parasites frequently involve the participation of glycoconjugates. * Trypanosoma brucei* expresses many glycoproteins containing Gal and GlcNAc, including glycoproteins with novel bloodstream form-specific giant poly-*N*-acetyllactosamine (poly-LacNAc) containing N-linked glycans (Mehlert, Richardson, et al. 1998; Mehlert, Zitzmann, et al. 1998; Atrih et al. 2005). The creation of UDP-glucose 4′-epimerase (*TbGalE*) conditional null mutants showed that this gene, and hence UDP-Gal, is essential for the survival of the parasite in both the bloodstream and procyclic life stages (Roper et al. 2002; Roper et al. 2005; Urbaniak et al. 2006). Similarly, the creation of UDP-GlcNAc pyrophosphorylase (*TbUAP*) and glucosamine 6-phosphate *N*-acetyltransferase (*TbGNA1*) conditional null mutants has shown that UDP-GlcNAc is also essential for bloodstream form *T. brucei* (Marino et al. 2011; Stokes et al. 2008). From these experiments, it is possible to conclude that one or more of the UDP-Gal- and UDP-GlcNAc-dependent glycosylation pathways are essential to the parasite. This has provided the impetus to identify and characterize the UDP-Gal and UDP-GlcNAc-dependent glycosyltransferase (GT) genes in the parasite. In a recent study, we mined the *T. brucei* genome for GTs and found a family of 21 genes with predicted amino acid sequences consistent with being UDP-sugar-dependent GTs.

The first of these genes to be studied were *TbGT8*, a UDP-GlcNAc:βGal β1-3 GlcNAc-transferase that modifies the complex GPI anchor side chains of the procyclins (Izquierdo, Nakanishi, et al. 2009) and also elaborates complex *N*-glycans in bloodstream from parasites (Nakanishi et al. 2014), and *TbGT11*, a UDP-GlcNAc:αMan β1-2 GlcNAc-transferase equivalent to GnTI and involved in the synthesis of complex *N*-glycan structures (Damerow et al. 2014).

In this work, we apply the methodology previously described for the analysis and phenotyping of GT mutants of *T. brucei* (Izquierdo, Nakanishi, et al. 2009; Izquierdo et al. 2013), to biochemically characterize another UDP-sugar-dependent GT, Tb927.2.3370 referred to here as *TbGT3*.

## Results

### Creation and analysis of a bloodstream form TbGT3 conditional null mutant

We selected a putative GT (Tb927.2.3370) from *T. brucei* to determine its activity by the creation and biochemical characterization of a conditional null mutant under nonpermissive conditions. The gene encodes a 377 amino acid protein, with a theoretical mass of 43.7 kDa. A semi-quantitative RT-PCR analysis previously performed over 21 full-length ORFs encoding putative UPD-Gal- or UDP-GlcNAc-dependent GTs in the *T. brucei* genome showed that the gene was present in both life cycle stages of the parasite, but more highly expressed in procyclic form trypanosomes (Izquierdo, Nakanishi, et al. 2009). This was confirmed in a recent quantitative proteomics study, which indicated that TbGT3 was expressed ∼2.5-fold higher in procyclic form trypanosomes at the protein level (Urbaniak et al. 2013).

The DXD sequence motif, common to many GTs (Wiggins and Munro 1998) and probably involved in binding a divalent cation, is present in the predicted protein as DDD, between amino acids 206 and 208. The protein has a predicted N-terminal transmembrane domain between residues 17–34 (Krogh et al. 2001) and is likely to be a type II membrane protein, typical for Golgi apparatus GTs (Colley 1997). The Tb927.2.3370 gene and 5′ and 3′ untranslated flanking sequences were amplified with a high-fidelity DNA polymerase from *T. brucei* strain 427 genomic DNA. Four clones from four separate PCR reactions were sequenced in both directions and the strain 427 sequence (Accession Number KF554011) was very similar to that in the strain 927 genome database. In the ORF, there were four apparent nucleotide polymorphisms and three of them produced amino acid changes: his225 in place of Tyr225, Gln269 in place of Glu269 and Arg301 in place of Gly301. The strain 427 gene and protein product will be referred to from here as *TbGT3* and TbGT3, respectively.

In order to analyze the function of the gene, we first created conditional null mutants in the bloodstream and procyclic forms of the parasite. A preliminary Southern blot analysis using a *TbGT3* ORF probe suggested that there is a single copy of the gene per haploid genome (Supplementary data, Figure S1), which was also consistent with BLAST search of the *T. brucei* genome database with the Tb927.2.3370 nucleotide sequence. Both *T. brucei TbGT3* alleles were replaced in bloodstream form parasites and an ectopic inducible copy of *TbGT3* under tetracycline control was introduced. To generate the conditional null mutants, genetically modified bloodstream form strains from 427 *T. brucei* containing a T7-RNA polymerase and a tetracycline repressor under the control of a T7 promoter were used (Wirtz and Clayton 1995). This cell line will be referred to as “wild type” from here on.

**Fig. 1.**
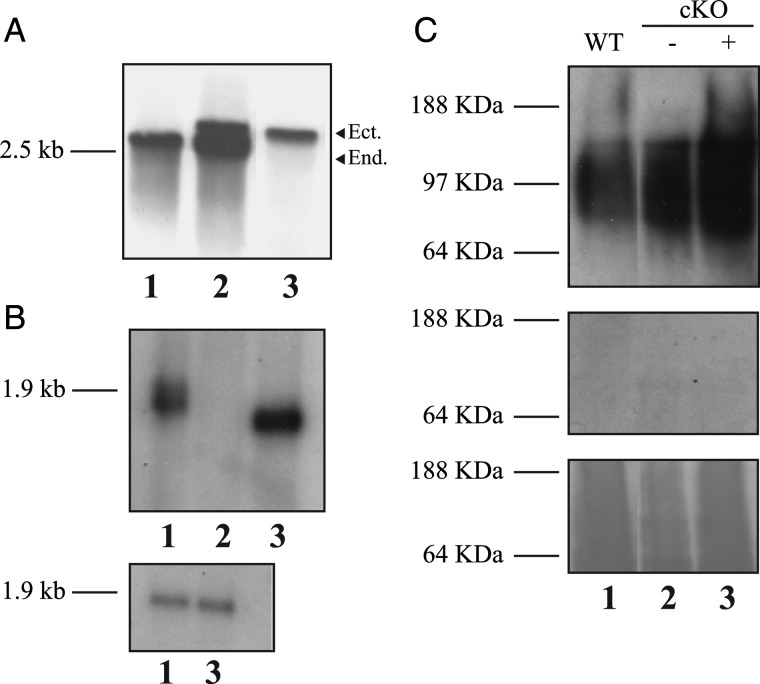
Characterization of *TbGT3* bloodstream form conditional null mutant. (**A**) Southern blot of genomic DNA from bloodstream form wild type (lane 1), wild-type + *TbGT3^Ti^* (lane 2) and *TbGT3* conditional null mutant (Δ*TbGT3*::*PAC*/*TbGT3^Ti^*/*ΔTbGT3*::*HYG*) (Lane 3) digested with EcoRI and probed with *TbGT3* ORF. The arrowheads indicate the bands corresponding to the endogenous (*End*) and ectopic (*Ect*) copies of the *TbGT3* gene. (**B**) Northern blot of total RNA extracted from wild-type cells (lane 1) and the *TbGT3* conditional null mutant grown in absence (lane 2) or presence of tetracycline (lane 3) for 48 h, probed with the *TbGT3* probe (upper panel) and the β-tubulin probe as a control (lower panel). (**C**) Blot of total glycoprotein extracts of wild type (lane 1), *TbGT3* conditional null mutant minus Tet (lane 2) and *TbGT3* conditional null mutant plus Tet (lane 3), incubated with WGA without (upper panel) and with inhibitors (specificity control, middle panel), and stained with pounceau (loading control, lower panel).

The first *TbGT3* allele was replaced in the bloodstream form by homologous recombination following electroporation of the parasites in the presence of linear DNA containing the puromycin acetyltransferase (*PAC*) drug-resistance gene flanked by ∼500 bp of *TbGT3* 5′- and 3′- untranslated region (UTR). Following selection with puromycin, a Δ*TbGT3*::*PAC* mutant was selected and transformed with an ectopic, tetracycline-inducible, copy of *TbGT3*, introduced into the ribosomal DNA locus under phleomycin selection (Wirtz et al. 1999). Then, maintaining tetracycline induction, the second endogenous allele was replaced by an hygromycin phosphotransferase (*HYG*) gene to yield the desired Δ*TbGT3*::*PAC*/*TbGT3^Ti^*/*ΔTbGT3*::*HYG* bloodstream form mutant. Southern analysis confirmed that both endogenous *TbGT3* gene copies were absent from the mutant (Figure 1A). Northern blot showed that *TbGT3* mRNA transcripts were absent under nonpermissive conditions (i.e., with no tetracycline added to the media) (Figure 1B). This and the normal in vitro growth kinetics of the *TbGT3* mutant in the absence of tetracycline (data not shown) allows us to conclude that *TbGT3* encodes a nonessential gene in bloodstream form *T. brucei*.

To observe any effects of the absence of TbGT3 activity on *T. brucei* bloodstream-form glycosylation, wild-type and *TbGT3* conditional null-mutant variant surface glycoprotein 221 (VSG221) was purified in a soluble form (Cardoso de Almeida and Turner 1983; Ferguson et al. 1985) from cells grown in the presence and absence of tetracycline for 48 h. Mature wild-type sVSG221 glycoprotein contains two N-glycosylation sites, one at Asn296 occupied by paucimannose and small complex structures and another at Asn428 occupied by oligomannose structures (Zamze et al. 1991), and a highly galactosylated GPI anchor glycan (Mehlert, Richardson, et al. 1998; Mehlert, Zitzmann, et al. 1998). Thus, we use the glycosylation status of sVSG221 as a convenient reporter to assess any effects that a mutation has on the formation of several glycosidic linkages (Jones et al. 2005; Urbaniak et al. 2006; Manthri et al. 2008; Izquierdo et al. 2012). Analysis of the glycoform pattern of sVSG221 from wild-type and *TbGT3* conditional null-mutant cells, grown in the presence and absence of tetracycline, by electrospray-mass spectrometry (ES-MS) did not reveal any alterations in its glycosylation pattern (data not shown).

In order to analyze the high-molecular-weight branched poly LacNAc-containing *N*-glycans of bloodstream form *T. brucei* (Atrih et al. 2005), we performed SDS/urea extraction of total glycoproteins from trypanosome ghosts (depleted of VSG) and western blotting with lectins. The extracts of wild-type and conditional cells before and after tetracycline induction were analyzed by ricin, which detects galactose residues, and wheat germ agglutinin (WGA) blotting. In the case of the ricin blot (data not shown), the patterns were similar to each other and to previous ricin blots (Urbaniak et al. 2006; Manthri et al. 2008; Stokes et al. 2008; Izquierdo, Nakanishi, et al. 2009). However, the absence of TbGT3 activity due to nonpermissive conditions very slightly decreased the binding of WGA (Figure 1C, see high-molecular-weight species). WGA has complex-binding properties, including binding to GlcNAcβ1-6Gal motifs (Muraki et al. 2002), which are characteristic of branched poly-*N*-acetyllactoaminoglycans previously described in blood stream trypanosomes. Thus, the differences observed suggest that the *TbGT3* product may play a minor role in the biosynthesis of branched poly-LacNAc structures in the bloodstream form of the parasite.

### Analysis of a procyclic form TbGT3 conditional null mutant under nonpermissive conditions

**Fig. 2.**
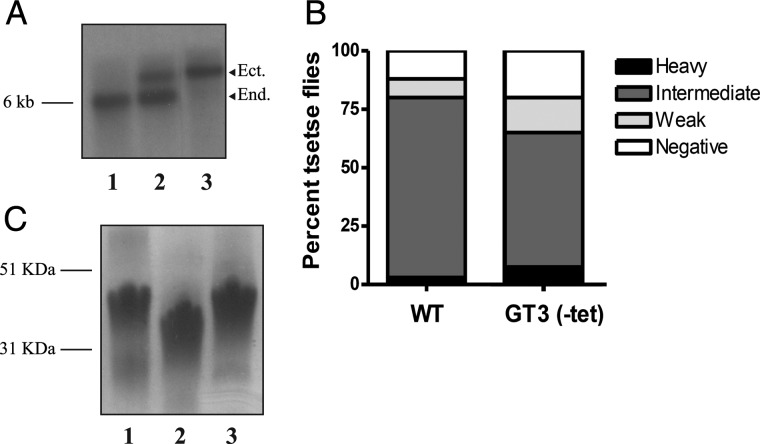
*TbGT3* conditional null-mutant procyclic cells grown in the absence of tetracycline are infective to tsetse flies and express smaller procyclin molecules than the wild type. (**A**) Southern blot of genomic DNA from procyclic form wild type (lane 1), wild-type + *TbGT3^Ti^* (lane 2) and *TbGT3* conditional null mutant (Δ*TbGT3*::*PAC*/*TbGT3^Ti^*/*ΔTbGT3*::*HYG*) (lane 3) digested with HindIII and probed with *TbGT3* ORF. The arrowheads indicate the bands corresponding to the endogenous (*End*) and ectopic (*Ect*) copies of the *TbGT3* gene. (**B**) Teneral tsetse flies were fed with bloodmeals containing either wild-type or GT3 null mutants in the absence of tetracycline (tet). After 22 days, the flies (40 in each group) were dissected and midgut infections scored as heavy (black; >100 trypanosomes/field), intermediate (dark gray; 20–100 trypanosomes/field), weak (light gray; 1–19 trypanosomes/field) or negative (no detectable cells). (**C**) Periodate-Schiff stained SDS–PAGE gel of extracted procyclins from wild type (lane 1) and *TbGT3* mutant cells grown without tetracycline (lane 2) or with tetracycline (lane 3).

In order to investigate if a more dramatic phenotype could be observed in the procyclic form of the parasite, a *TbGT3* conditional null mutant was prepared in this stage of the parasite using cell line 29.13.6 (derived from *T. brucei* strain 427), that retain genomic T7-RNA polymerase and TETR genes in presence of G418 and hygromycin selection (Wirtz et al. 1999). The *TbGT3* alleles were replaced using the same targeting vectors used in the bloodstream form of the parasite, but containing *PAC* and blasticidin S deaminase (*BSD*) drug-resistance cassettes and an ectopic, tetracycline-inducible, copy of *TbGT3*, introduced into the ribosomal DNA locus under phleomycin selection increasing the number of *TbGT3* genomic copies (Wirtz et al. 1999). The glycosylation phenotype was assessed in this *TbGT3* procyclic form conditional null mutant, Δ*TbGT3*::*PAC*/*TbGT3^Ti^*/*ΔTbGT3*::*BSD*, after confirmation by Southern blot that both *TbGT3* gene endogenous copies were absent in the mutant (Figure 2A). As in the case of the bloodstream form, procyclic form conditional null mutants were able to grow normally in vitro without the presence of tetracycline in the media, showing that the gene is also nonessential in the procyclic form of the parasite. Furthermore, mutants grown in nonpermissive conditions were able to colonize the tsetse midgut, although the infectivity was marginally reduced compared with that of the wild type (Figure 2B). To assess the glycosylation phenotype of procyclic form parasites, procyclins from wild-type and conditional null mutant grown in the absence or presence of tetracyclin were extracted (Ferguson et al. 1993; Izquierdo et al. 2013) and analyzed by SDS–PAGE and periodate-Schiff staining for carbohydrate. This revealed a significant reduction in the apparent molecular weights of the *TbGT3* null-mutant procyclins grown in the absence of tetracycline in the media for 48 h compared with wild type and the mutant grown in the presence of tetracycline (Figure 2C). Thus, the reduction in procyclin apparent molecular weight is specifically due to the lack of expression of the *TbGT3* gene.

**Fig. 3.**
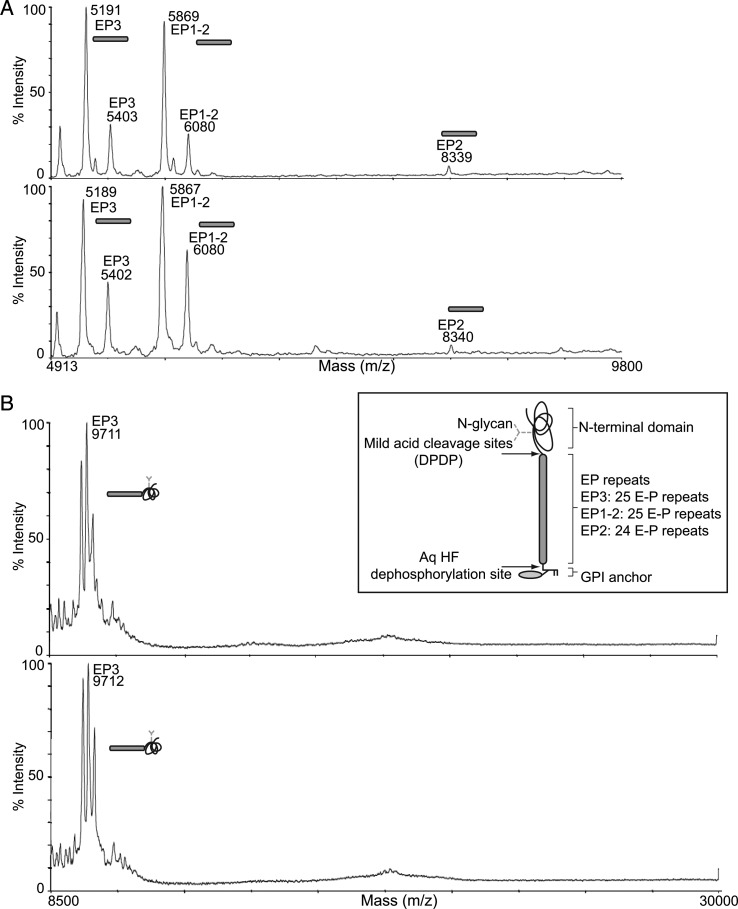
MALDI-ToF mass spectra of procyclins from the *TbGT3* conditional null mutant show no differences in procyclin polypeptide or N-glycosylation structure. (**A**) MALDI-ToF mass spectra of aq. HF dephosphorylated and mild acid-treated procyclins from the *TbGT3* conditional null mutant grown in absence (top panel) or presence of tetracycline (bottom panel) for 48 h. (**B**) MALDI-ToF mass spectra of aq. HF dephosphorylated procyclins from the *TbGT3* conditional null mutant grown in absence (top panel) or presence of tetracycline (bottom panel) for 48 h. The diagnostic peptide ions (Acosta-Serrano et al. 1999) for the different types of procyclins are indicated.

The procyclin polypeptides extracted from the *TbGT3* mutant cells in absence and presence of tetracycline were analyzed by MALDI-ToF after aq. HF dephosphorylation with and without mild acid treatment (Acosta-Serrano et al. 1999; Mehlert et al. 1999). The dual aq. HF dephosphorylation with mild acid treatment cleaves off the GPI anchors through dephosphorylation and cleaves the very acid-labile Asp-Pro (DP) peptide bonds present on the N-terminal side of EP-repeats of the EP-procyclins. This yields N-glycosylated peptide fragments that are characteristic of the procyclin types being expressed, in this case a mixture of EP3, EP1-2 and a trace of EP2 (Figure 3A). When the mild acid hydrolysis step is omitted, the fragments generated by GPI anchor removal alone still contain the N-glycosylated N-terminal domains of the procyclins (Figure 3B). In this case, we can see ions corresponding to the EP3 and EP1-2 protein components bearing Hex_5_HexNAc_2_ *N*-glycan structures (Acosta-Serrano et al. 1999). Taken together, these analyses show that the reductions in the apparent molecular weights of the *TbGT3* conditional null mutant grown in the absence of tetracycline were most likely due to alterations in the structure of the GPI anchor side chains (Izquierdo, Nakanishi, et al. 2009) since, upon GT3 deletion, the repertoire of procyclin proteins and the size of their *N*-glycans remained unchanged.

### Characterization of the changes in the procyclin GPI anchor side chain in the TbGT3 conditional null mutant under nonpermissive conditions

**Fig. 4.**
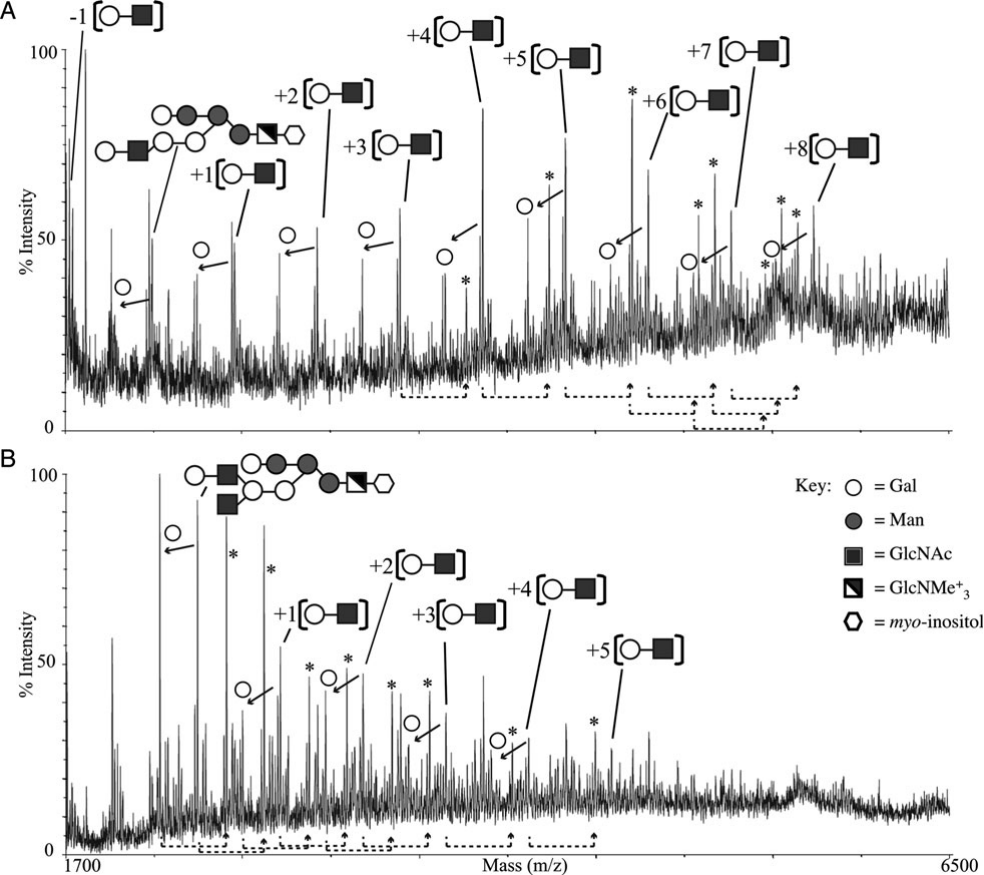
MALDI-ToF mass spectra of permethylated GPI glycans isolated from wild type and *TbGT3* conditional null-mutant trypanosomes grown in absence of tetracycline. Permethylated glycans of the GPI anchors of wild type (**A**) and *TbGT3* conditional null mutant grown in absence of tetracycline (**B**) were analyzed by positive MALDI-ToF MS after co-crystallization with a 2,5-dihydroxybenzoic acid matrix. A series of GPI glycans with several Hex-HexNAc repeats are indicated. Open circles show a similar series minus a terminal Hex residue, presumably attached to the mannose core (solid arrows indicate neutral losses of 204 Da, equivalent to one methylated hexose residue). Asterisks indicate molecules with sialic acid residues (up to three sialic acid residues can be detected in the wild-type GPI anchors; dotted arrows indicate 361 Da mass increases, equivalent to methylated sialic acid residues).

In order to analyze the specific changes in procyclin GPI anchor side chain structure induced by the lack of *TbGT3* expression, procyclins were isolated from wild-type and *TbGT3* conditional null mutant grown in the absence of tetracycline. GPI anchor glycans were released from the procyclin peptide and the *lyso*-phosphatidic acid lipid component of the PI moiety by aq. HF dephosphorylation (a procedure that also removes some sialic acid residues). The released GPI glycans were subsequently permethylated, a procedure that removes the fatty acid from the inositol ring, methylates all free hydroxyl groups and converts the amine group of the glucosamine residue to a positively charged quaternary amine (Baldwin 2005; Mehlert and Ferguson 2007; Izquierdo, Nakanishi, et al. 2009; Nett et al. 2010). Analysis of the permethylated GPI glycan fractions by positive ion MALDI-ToF produced complex spectra (Figure 4). The [M]^+^ molecular ions in the spectra can be accounted for by assuming core structures of Hex_6_HexN(Me_3_)^+^Ino substituted with between zero and eight HexHexNAc units and with the presence of sialic acid residues in some cases (Figure 4A). Although not a quantitative technique, the glycan species from the *TbGT3* conditional null mutant grown under nonpermissive conditions appear to be significantly smaller (Figure 4B). Series from Hex_5_HexN(Me_3_)^+^Ino, most probably from Hex_6_HexN(Me_3_)^+^Ino losing a terminal galactose residue, can also be observed in panels A and B (see the open circles plus arrows).

**Fig. 5.**
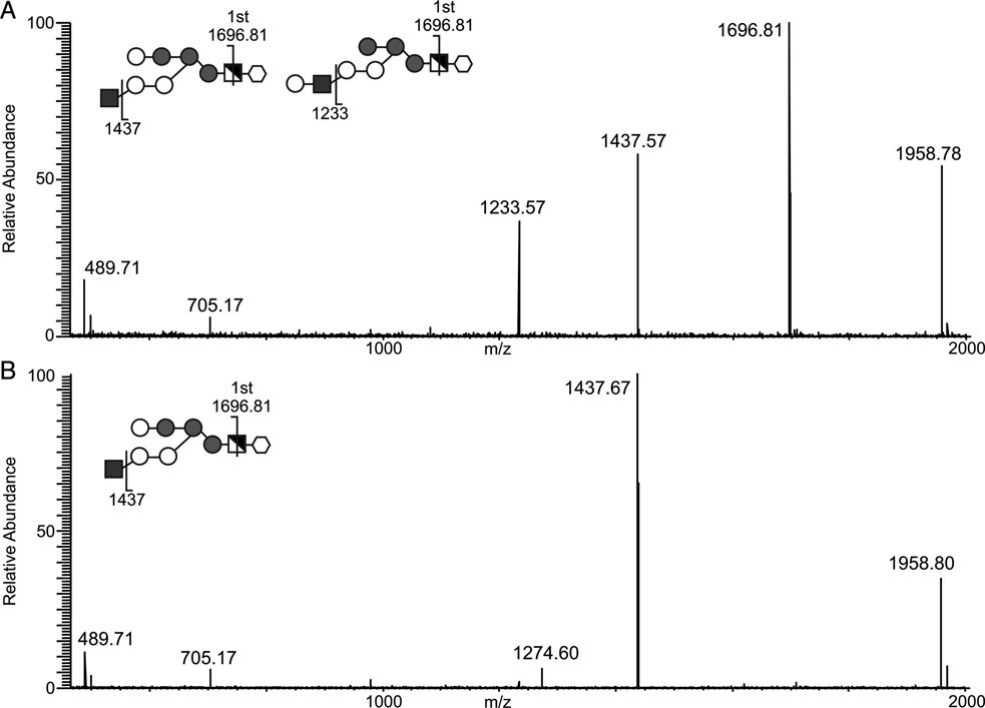
MS^3^ product ion spectra of permethylated GPI glycans show the presence of a terminal Hex-HexNAc unit in the wild type and a terminal HexNAc in the *TbGT3* conditional null mutant grown in absence of tetracycline. Permethylated glycans of the GPI anchors of wild type (**A**), *TbGT3* conditional null mutant grown in absence tetracycline (**B**) were analyzed by positive ESI-MS. Doubly charged [M+Na]^2+^ ions observed at *m*/*z* 994.51 were fragmented (MS^2^) generating a singly charged daughter ion at *m*/*z* 1697.99. This ion was further fragmented (MS^3^) to generate the product ion spectra shown in the figure. Assignments of the diagnostic ions are indicated on the inset diagrams. The product ion at *m*/*z* 1437 is diagnostic of the presence of a t-GlcNAc in the Hex6HexN(Me3)^+^Ino core structure (both panels), whereas the product ion at *m*/*z* 1233 (only in panel A) is diagnostic of the presence of a HexHexNAc unit attached to a Hex5HexN(Me3)^+^Ino core. The key is the same as in Figure 4.

The same permethylated GPI glycan samples were also analyzed by positive ion ESI-MS and doubly charged [M+Na]^2+^ ions of (HexNAc)Hex_6_HexN(Me_3_)^+^Ino species were observed at *m*/*z* 994.51. Fragmentation of these ions (MS^2^) produced an intense singly charged product ion at *m*/*z* 1696.81, arising from elimination of the inositol residue and quaternary amine group (Supplementary data Figure S2), which on further fragmentation (MS^3^) produced the product ion spectra shown in Figure 5. The spectra show differences in the compositions of the isobaric permethylated glycans between the two samples. The wild-type sample contains two principle components, (HexNAc)Hex6HexN(Me_3_)^+^Ino and (HexHexNAc)Hex_5_HexN(Me_3_)^+^Ino (Figure 5A), whereas the *TbGT3* mutant only contains the (HexNAc)Hex_6_HexN(Me_3_)^+^Ino species (Figure 5B). These data strongly suggest that *TbGT3* encodes a Gal transferase that acts on the GPI anchor side chain to add a Gal residue to the non-reducing-terminal GlcNAc residue.

**Fig. 6.**
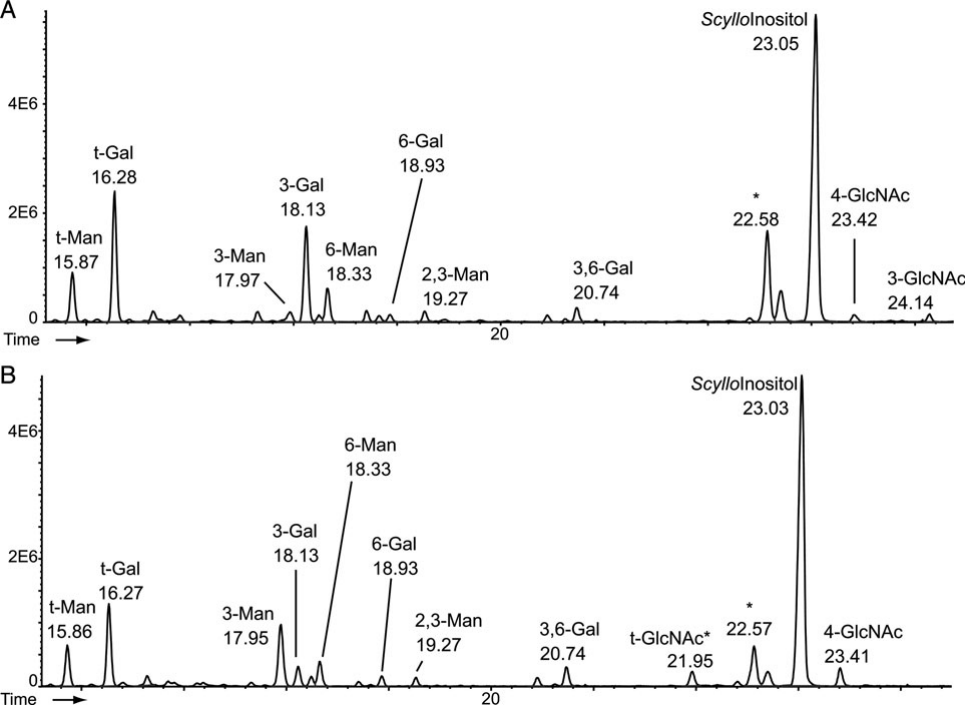
GC–MS linkage analysis of permethylated GPI glycans reveals differences between the wild type and the *TbGT3* conditional null mutant grown under nonpermissive conditions. Permethylated glycans of the GPI anchors of wild type (**A**) and *TbGT3* conditional null mutant grown in absence of tetracycline (**B**) were acid hydrolyzed, reduced, acetylated and the resulting PMAA derivatives were analyzed by GC–MS to produce the total ion chromatograms shown. The peak marked *scyllo*-inositol is an internal standard of *scyllo*-inositol hexa-acetate. The peak marked with an asterisk is a reagent artefact. The PMAA peaks are annotated according to the original substitution pattern of the native glycans. For example, t-Man refers to non-reducing-terminal mannose and 3-Man refers to 3-O-substituted mannose (see Supplementary data Table S1). The peak marked t-GlcNAc* in (B) is a mixture of t-GlcNAc PMMA and non-sugar contaminant.

The same permethylated GPI glycan fractions were subjected to acid hydrolysis, reduction and acetylation to provide partially methylated alditol acetate derivatives (PMAAs) that were analyzed by GC–MS to provide a methylation linkage analysis (Figure 6; Supplementary data, Table S1). Normalizing the PMAA signals to the 6-*O*-substituted Man residue of the conserved Man_3_GlcN-Ino core, we can see that the *TbGT3* mutant GPI glycan is deficient in 3-*O*-substituted Gal residues and does not contain any 3-*O*-substituted GlcNAc residues, whereas wild-type GPI glycans contain both 4- and 3-*O*-substituted GlcNAc. Thus, the methylation linkage data suggest that TbGT3 is a β1-3 Gal-transferase that acts on one of the GlcNAc residues of the GPI anchor side chain. Consistent with this, some non-reducing-terminal GlcNAc (t-GlcNAc) is also apparent in the methylation linkage analysis (Figure 6B). Interestingly, these are not the only changes of PMAAs relative to 6-*O*-substituted Man; there are significant increases in 4-*O*-substituted GlcNAc and 3,6-di-*O*-substituted Gal residues, suggesting that there may be some compensatory mechanism at work in the absence of TbGT3 activity. This could include, for example, the modification of some of the βGlcNAc normally used as the acceptor substrate by TbGT3 by a β1-4 Gal-transferase.

## Discussion

The survival strategies of protozoan parasites frequently involve the participation of glycoconjugates. In the particular case of *T. brucei*, several precursors required for the biosynthesis of these glycoconjugates such as UDP-Gal and UDP-GlcNAc (Roper et al. 2002, 2005; Stokes et al. 2008; Marino et al. 2011) are essential for the survival of the parasite in the bloodstream and/or procyclic form life stages. This prompted us to mine the *T. brucei* genome for UDP-sugar-dependent GTs and to investigate their essentiality, function and specificity by the creation of null or conditional null mutants. The fact that the *TbGT3* conditional null-mutant cell lines grow normally in vitro in the absence of tetracycline in both the procyclic and the blood stream form of the parasite, show that TbGT3 is nonessential for the survival of *T. brucei*. Similarly, experiments carried out in mice and tsetse flies indicate that the gene is nonessential for the survival of the parasite in vivo, though of course these types of experiment generally fail to pick up subtle fitness traits that can be sufficient to maintain the genes in nature.

Using a well-established workflow for the functional and biochemical characterization of *T. brucei* GTs (Izquierdo, Nakanishi, et al. 2009; Izquierdo et al. 2013), we designate TbGT3 as primarily a GPI side chain modifying UDP-Gal : βGlcNAc β1-3 Gal-transferase. However, like TbGT8, which is both a GPI side-chain modifying UDP-GlcNAc:βGal β1-3 GlcNAc-transferase (Izquierdo, Nakanishi, et al. 2009) and involved in large complex *N*-glycan synthesis in bloodstream form parasites (Nakanishi et al. 2014), TbGT3 is likely also to be involved in some way in the synthesis of large complex *N*-glycans in the bloodstream form of the parasite (Atrih et al. 2005; Izquierdo, Nakanishi, et al. 2009).

**Fig. 7.**
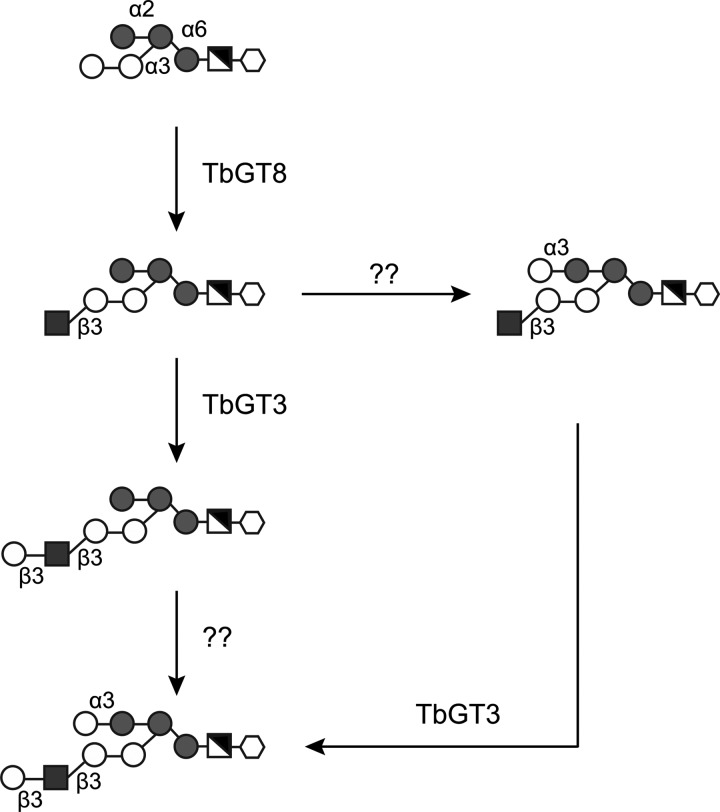
Model of GPI anchor side-chain biosynthesis initiation in procyclic form trypanosomes. The first branching point in the GPI anchor side-chain is produced by TbGT8, a GlcNAc-transferase that acts on the terminal digalactose moiety of the GPI anchor (Izquierdo, Nakanishi, et al. 2009; Nakanishi et al. 2014). The resulting GPI structure can be further substituted either by (i) TbGT3 Gal-transferase, described in this paper, that adds a Gal residue to the non-reducing–t-GlcNAc or (ii) an unknown Gal-transferase, labelled as “??”, that adds a Gal-terminal residue to the α1,2-linked mannose.

With respect to the GPI anchor modifying activity of TbGT3, we postulate that it acts on the product of TbGT8, either directly or after the action of an unknown α1-3 Gal-transferase (Figure 7).

## Materials and methods

### Cultivation of trypanosomes

Bloodstream form *T. brucei* genetically modified to express T7 polymerase and the tetracycline repressor protein were cultivated in HMI-9 medium containing 2.5 μg mL^−1^ G418 at 37°C in a 5% CO_2_ incubator as described in Wirtz et al. (1999). *Trypanosoma brucei* procyclic form parasites that maintain T7 polymerase and tetracycline repressor protein under G418 and hygromycin selection (cell line 29:13) (Wirtz et al. 1999) were cultured in SDM-79 medium at 28°C.

### DNA isolation and manipulation

Plasmid DNA was purified from *Escherichia coli* (DH5α) using Qiagen Miniprep or Maxiprep kits, as appropriate. Gel extraction was performed using Qiaquick kits. Custom oligonucleotides were obtained from the Dundee University oligonucleotide facility. *Trypanosoma brucei* genomic DNA was isolated from∼2 × 10^8^ bloodstream form cells or from 1 × 10^9^ procyclic cells using DNAzol (Helena Biosciences).

### Generation of constructs

The 413 bp 5′- and 401 bp 3′-UTR sequences next to the Tb927.2.3370 ORF were PCR-amplified from genomic DNA using Platinum Taq DNA polymerase HiFi (Invitrogen) with 5′-atgGCGGCCGCgatgaggaaaggttgctgagtt-3′ and 5′-gtttaaacttacggaccgtcaagctttttccggatcgtatacctaaaac-3′ and 5′- gacggtccgtaagtttaaacggatcctttaggctccaattggtaaaag -3′ and 5′-gatGCGGCCGC gtaaacacacacgagaaaacaaca-3′ as forward and reverse primers, respectively. The two PCR products were used together in a further PCR reaction to yield a product containing the 5′-UTR linked to the 3′-UTR by a short HindIII and BamHI cloning site (underlined) and NotI restriction sites at each end (capital letters). The PCR product was cloned into the NotI site of the pGEM-5Zf(+) vector (Promega) and the HYG and PAC drug-resistance genes were introduced into the targeting vector via the HindIII and BamHI cloning sites. For re-expression of Tb927.2.3370 the ORF was PCR amplified from genomic DNA using Platinum Taq DNA polymerase HiFi with 5′-cgcAAGCTTatggtgtcgaagggtttac-3′ and 5′-cgcGGATCCtcagcatttcccacgagc-3′ and cloned into the pLew100 vector (Wirtz et al. 1999) cut with HindIII and BamHI, to generate pLewGT3. This plasmid was then digested with NotI, and purified for transfection.

### Transformation of bloodstream and procyclic form *T. brucei*

Constructs for gene replacement and ectopic expression were purified using the Qiagen Maxiprep kit, digested with NotI to linearize, precipitated and washed twice with 70% ethanol and re-dissolved in sterile water. Cell culture and transformation were carried out as previously described (Wirtz et al. 1999; Milne et al. 2001).

### Southern and Northern blotting

Aliquots of genomic DNA isolated from *T. brucei* cultures were digested with various restriction enzymes. Fluorescein-labelled probes were generated using the CDP-star random prime labelling kit (Gene Images); 250 ng of template was used in a reaction volume of 50 μL and incubated for 90 min. Aliquots of 5 μL were used for each Southern blot experiment.

Total RNA for Northern blots was prepared using Qiagen RNeasy Midi kits. Samples of RNA (5 μg) were run on formaldehyde-agarose gels and transferred to Hybond N nylon membrane (Amersham Biosciences) for hybridization with [α-^32^P]-dCTP-labeled *T. brucei* Tb927.2.3370 probe (Stratagene, Prime-It RmT random primer labelling kit). The control β-tubulin probe was used after the Tb927.2.3370 probe had decayed.

### Tsetse fly infections

Tsetse fly infections were carried out as described in Guther et al. (2006). Pupae of *Glossina morsitans morsitans* were obtained from Institute of Zoology, Slovak Academy of Science (Bratislava, Slovakia) and maintained at the WCMP tsetse fly facility, University of Glasgow. Newly hatched (teneral) flies were fed with an infected bloodmeal, which consisted of 10^7^ parasites mixed with washed defibrinated horse blood (containing 10% fetal bovine serum). Infected flies were fed with bloodmeals every 2–3 days. After 2 weeks or 3 weeks, midguts were isolated from infected flies and disrupted by mechanical force in cold SDM-79 containing 10% fetal bovine serum. Isolated parasites from individual midguts were kept on ice until counted on an hemocytometer.

### Purification of procyclins

Procyclins (both GPEET and EP forms) were purified from 10^11^ freeze dried trypanosomes by organic solvent extraction followed by octyl-Sepharose chromatography (Amersham Pharmacia Biotech) (Ferguson et al. 1993; Treumann et al. 1997; Mehlert et al. 1999). For some specific procedures, such as SDS–PAGE and periodate-Schiff stain, procyclins were extracted from batches of ∼1 × 10^8^ cells and were run directly with the extracts without prior purification by octyl-Sepharose chromatography.

### Analysis by MALDI-ToF MS

Approximately 250 pmol of octyl-Sepharose-purified procyclins determined by GC–MS (Ferguson 1994) was dried and treated with 50 μL of ice-cold 50% aqueous hydrogen fluoride for 24 h at 0°C to cleave the GPI anchor ethanolamine-phosphate bond. Some preparations were further treated with 50 μL of 40 mM trifluoroacetic acid, 100°C for 20 min to cleave Asp-Pro bonds and remove N-glycosylated N termini (Acosta-Serrano et al. 1999). The samples were dried and re-dissolved in 5 μL of 0.1% trifluoroacetic acid. Aliquots (1 μL) of each sample were mixed with 1 μL of 10 mg mL^−1^ sinapinic acid in 50% acetonitrile, 0.1% trifluoroacetic acid and analyzed by positive ion MALDI-ToF. Data collection was in linear mode on a Voyager-DE STR instrument. The accelerating voltage was 25,000 V, and grid voltage was set at 94% with an extraction time delay of 700 ns. Data were collected manually at 500 shots/spectrum with the laser intensity set at 2500. The analysis of the native procyclins (500 pmol/sample) was performed in negative mode using sinapinic acid as the matrix. For the analysis of the permethylated GPI glycans, the instrument was set in reflectron mode, positive ion and a matrix of 2,5-dihydroxybenzoic acid (15 mg mL^−1^ in 0.5% TFA) was used.

### Permethylation and ES-MS of GPI glycans

Samples were dried and permethylated by the sodium hydroxide and methyl iodide method, as described in Ferguson (1994). The permethylated glycans were dissolved in 50 μL of 80% acetonitrile, and aliquots (5 μL) were dried and recovered in 80% acetonitrile, 0.5 mM sodium acetate before loading into nanotips (Micromass type F) for positive ion ES-MS, ES-MS2 and ES-MS3 on an LTQ Orbitrap Velos mass spectrometer (Thermo Scientific). Source and capillary voltages were 0.63 kV and 48 V, respectively, and the collision energy was 17–19%.

### Methylation linkage analysis by GC–MS

The remainder of the permethylated glycan samples were subjected to acid hydrolysis, NaB^2^H_4_ reduction and acetylation (to yield PMAAs), and analyzed by GC–MS as described in Ferguson (1994). The PMAAs were analyzed on an Agilent 6890 N GC–MS system fitted with an HP-5 column.

### Lectin blotting of *T. brucei* cell extracts

Bloodstream form *T. brucei* cells were washed twice in trypanosome dilution buffer (5 mM KCl, 80 mM NaCl, 1 mM MgSO_4_, 20 mM Na_2_HPO_4_, 2 mM NaH_2_PO_4_, 20 mM glucose, pH 7.4), solubilized with 2% SDS and 4 M urea, subjected to SDS–PAGE and transferred to nitrocellulose membranes (Amersham Biosciences). The membranes were incubated for 1 h with biotin-conjugated ricin (Vector Labs) diluted 1/3000, with or without inhibitors (10 mg mL galactose and 10 mg mL lactose) or 1 h with biotin-conjugated WGA (Vector Laboratories) diluted 1/2000, with or without inhibitor (1/10 Chitin hydrolysate). Membranes were then incubated for 1 h with extravidin-HRP diluted 1/10000, and developed with ECL (Amersham Biosciences) according to the manufacturer's instructions.

## Supplementary data

Supplementary data for this article are available online at http://glycob.oxfordjournals.org/.

## Conflict of interest statement

None declared.

## Funding

M.A.J.F. and A.M. are supported by a Wellcome Trust Senior Investigator Award (101842). A.A.-S. is supported by a Wellcome Trust grant (WT093691MA). L.I. is supported by the Ramón y Cajal Program (Spanish Ministry of Economy and Competitiveness). M.A.J.F., L.I. and A.A.-S. are members of the GlycoPar-EU FP7 funded Marie Curie Initial Training Network. Mass spectrometry was performed in the Fingerprints Proteomics Facility at The University of Dundee, supported by Wellcome Trust Strategic Award 097945 and we thank Douglas Lamont for assistance. Funding to pay the Open Access publication charges for this article was provided by The Wellcome Trust.

## Abbreviations

*BSD*, blasticidin S deaminase; ES-MS, electrospray-mass spectrometry; GPI, glycosylphosphatidylinositol; GT, glycosyltransferase; *HYG*, hygromycin phosphotransferase; PMAAs, partially methylated alditol acetate derivatives; *PAC*, puromycin acetyltransferase; t-GlcNAc, terminal GlcNAc; UTR, untranslated region; VSGs, variant surface glycoprotein; VSG221, variant surface glycoprotein 221; WGA, wheat germ agglutinin.

## Supplementary Material

Supplementary Data
