# Supplementary material for: Identification of a glycosylphosphatidylinositol anchor-modifying β1-3 galactosyltransferase in Trypanosoma brucei
Source: Glycobiology. 2014 Dec 2;25(4):438–47. doi: 10.1093/glycob/cwu131 (PMC4339879; doi:10.1093/glycob/cwu131)
Supplement: Supplementary Data [file supp_25_4_438__index.html]

Identification of a glycosylphosphatidylinositol anchor-modifying β1-3 galactosyltransferase in Trypanosoma brucei — Identification of a glycosylphosphatidylinositol anchor-modifying β1-3 galactosyltransferase in Trypanosoma brucei — Identification of a glycosylphosphatidylinositol anchor-modifying β1-3 galactosyltransferase in Trypanosoma brucei — Supplementary Data 

# Identification of a glycosylphosphatidylinositol anchor-modifying β1-3 galactosyltransferase in *Trypanosoma brucei*

## Supplementary Data

Supplementary Data

**Files in this Data Supplement:**

- Supplementary Data - Doc file
