# Supplementary material for: Identification of a glycosylphosphatidylinositol anchor-modifying β1-3 galactosyltransferase in Trypanosoma brucei
Source: Glycobiology. 2014 Dec 2;25(4):438–47. doi: 10.1093/glycob/cwu131 (PMC4339879; doi:10.1093/glycob/cwu131)
Supplement: Supplementary Data [file supp_cwu131_cwu131supp.doc]

TABLE SI

GC-MS methylation linkage analysis of GPI glycans from wild type and *TbGT3* conditional null mutant grown in absence and presence of tetracyclin procyclins.

The purified GPI anchors were permethylated, hydrolyzed, deutero-reduced, and acetylated to yield PMAAs for analysis by GC-MS. Residue types were deduced from the electron-impact mass spectra and retention times. ND, not detected

| PMAA | Residue type | Rt*^a^* | Sample*^b^* | |
| --- | --- | --- | --- | --- |
|  |  |  | Wild type | *TbGT3* - Tet |
| [1-^2^H]-1,5-Di-*O*-acetyl-2,3,4,6-tetra-*O*-methyl-mannitol | t-Man | 15.86 | ++ | ++ |
| [1-^2^H]-1,3,5-Tri-*O*-acetyl-2,4,6-tri-*O*-methyl-mannitol | 3-Man | 17.96 | + | ++ |
| [1-^2^H]-1,5,6-Tri-*O*-acetyl-2,3,4-tri-*O*-methyl-mannitol | 6-Man | 18.33 | + | + |
| [1-^2^H]-1,2,3,5-Tetra-*O*-acetyl-4,6-di-*O*-methyl-mannitol | 2,3-Man | 19.27 | + | + |
| [1-^2^H]-1,5-Di-*O*-acetyl-2,3,4,6-tetra-*O*-methyl-galactitol | t-Gal | 16.27 | +++ | +++ |
| [1-^2^H]-1,3,5-Tri-*O*-acetyl-2,4,6-tri-*O*-methyl-galactitol | 3-Gal | 18.13 | +++ | + |
| [1-^2^H]-1,5,6-Tri-*O*-acetyl-2,3,4-tri-*O*-methyl-galactitol | 6-Gal | 18.93 | + | + |
| [1-^2^H]-1,3,5,6-Tetra-*O*-acetyl-2,4-di-*O*-methyl- galactitol | 3,6-Gal | 20.74 | + | + |
| [1-^2^H]-1,5-Di-*O*-acetyl-3,4,6-tri-O-methyl-2-methylacetamido-glucosaminitol | t-GlcNAc | 21.95 | ND | + |
| [1-^2^H]-1,3,5-Tri-*O*-acetyl-2-methylacetamido-4,6-di-*O*-methyl-glucosaminitol | 3-GlcNAc | 24.14 | + | ND |
| [1-^2^H]-1,4,5-Tri-*O*-acetyl-2-methylacetamido-3,6-di-*O*-methyl-glucosaminitol | 4-GlcNAc | 23.42 | + | + |

*^a^* Retention time, in minutes

*^b^* +++ peak area >10^6^; ++ peak area between 5x10^5^-10^6^; + peak area <5x10^5^
